# Understanding the contribution of intellectual disabilities nurses. Paper 4 of 4 - Impacts of intellectual disability nursing interventions

**DOI:** 10.1177/17446295241228044

**Published:** 2024-01-18

**Authors:** Kay Mafuba, Hazel M Chapman, Rebecca Chester, Joann Kiernan, Chiedza Kudita, Dorothy Kupara

**Affiliations:** 7364 University of West London, UK; 11965 University of Chester, UK; 1900 Berkshire Healthcare NHS Foundation Trust, UK; 6249 Edge Hill University and Alder Hey Children’s Hospital, UK; 7364 University of West London, UK

**Keywords:** intellectual disability, nursing intevensions, impact

## Abstract

Internationally, there is a wide variety of roles and expectations for intellectual disabilities nurses, and the range of nursing interventions they undertake in this field has not been clearly identified. In this paper we report the impacts of intellectual nursing interventions from an online survey of intellectual disability nurses. An online survey, using voluntary response sampling was used to collect case study examples from 230 participants from seven countries. We identified 13 themes of the impacts, and 23 broad groups of case examples of intellectual disability nursing interventions with, pregnant women, children, adults, older adults, and people at the end of life. Awareness of the roles of intellectual disability nurses and their importance in addressing health inequalities and facilitating the use of mainstream services for people with intellectual disabilities will enable improved healthcare experience and healthcare outcomes for people with intellectual disabilities.

## Introduction and background

This is part 4 of a 4-part series. The overall aim of the research was to identify nursing led interventions that are in place to address the challenging and changing needs of people with intellectual disability. In addition, the research sought to investigate the impacts of intellectual disability nursing intervention on health outcomes for people with intellectual disabilities. Paper 1 reports the findings from the scoping literature review that was undertaken between 1 February 2020 and 31 May 2020. Paper 2 reports the findings from an online cross-sectional survey of intellectual disability nurses that identified 5 major themes of nursing interventions: effectuating nursing procedures, enhancing impact of intellectuality disability services, enhancing impact of mainstream services, enhancing quality of life, and enhancing intellectual disability nursing practice. Paper 3 reports the findings from evaluation questions of an online survey of intellectual disability and other nurses working with people with intellectual disabilities of how they understood these interventions. The objective of this part of the study was to a evaluate intellectual disability nurses’ confidence in their understanding of the interventions they undertook. We also sought to understand the factors that influenced interventions undertaken by these nurses within diverse settings across the lifespan. In this paper, paper 4, we report the impacts of intellectual disability nursing interventions and other nurses working predominantly with people with intellectual disabilities.

Approximately 1.5 million people (2.16% of adults and 2.5% of children) in the UK, are identified as having an intellectual disability (Mencap, 2020). Despite avoidable disparities in health between people with intellectual disability and the general population (Kerr, 2004; van Schrojenstein Lantman-de Val et al., 2007), their life expectancy is increasing, along with the complexity of their health and social care needs (Truesdale and Brown, 2017). However, there is a wide variety of roles and expectations for intellectual disability nurses globally. The impact of these roles and interventions has not been clearly identified.

People with intellectual disability have much greater health needs than others (Backer et al., 2009), and experience preventable higher mortality rates (LeDeR, 2020). They are often or more likely to be dependent on others for their health and healthcare outcomes (Campbell and Martin, 2009). Oullette-Kuntz (2005) concluded that healthcare outcomes for people with intellectual disability could be improved through appropriate intellectual disability nursing interventions. Given that people with intellectual disability are high and frequent users of all health and social care services, the positive impacts of intellectual disability nursing interventions need to be clearly described.

There are longstanding and widespread concerns about the inequalities in health, and poor access to healthcare for people with intellectual disability (Kavanagh et al., 2017; Brown et al., 2010; Melville et al., 2006). The barriers to health services experienced by people with intellectual disability contribute to the health inequalities they live with. The lack of clarity of what intellectual disability nurses do to address these barriers and inequalities has been identified as one of the barriers (Mafuba, 2009, 2013; Mafuba and Gates, 2015; Mafuba et al., 2018). intellectual disability nurses have an important role in meeting the health needs of people with intellectual disability but there is a lack of evidence of the impacts of their interventions.

Northway et al. (2017) suggested that intellectual disability nurses can intervene by being involved in pre-natal screening and providing support in relation to diagnosis. Intellectual disability nurses play important roles in promoting the health and wellbeing of children with intellectual disability (Delahunty, 2017; Northway et al., 2017). Some of the interventions identified in existing studies include educating healthcare professionals about the needs of people with intellectual disabilities needing end of life care (Dalgarno and Riordan, 2014); educating healthcare professionals about the needs of people with intellectual disabilities needing end of life care (Lee and Kiemle, 2014); preparing women psychologically for cancer screening (Lloyd and Coulson, 2014); record keeping (Lovell et al., 2014); making and facilitating reasonable adjustments (MacArthur et al., 2015); advocating for people with intellectual disabilities and / or their families (Brown et al., 2016); promoting independence (Drozd and Clinch, 2016); supporting people with intellectual disabilities with a history of offending behaviour to develop appropriate relationships (Lovell and Bailey, 2016); advocating for people with intellectual disabilities and / or their families (Ring et al., 2018); and assessing risk (Pennington et al., 2019). Studies have demonstrated that intellectual disability nurses work with older adults (Jenkins, 2012; Doody et al., 2013; Auberry and Cullen, 2016; Nelson and Carey, 2016; Cleary and Doody, 2017), as well as with people with intellectual disability receiving palliative care (McCarron et al., 2018; Wagemans et al., 2015; Ng, 2011). However, what is not clear in the studies, and what this paper addresses are the impacts of these interventions on the health and wellbeing of people with intellectual disabilities.

In addition, existing studies have demonstrated that intellectual disability nurses play a significant role in enhancing the effectiveness of health interventions undertaken by other professionals (Drozd and Clinch, 2016; Marriott et al., 2015; Lloyd and Coulson, 2014). MacArthur et al. (2015) concluded that intellectual disability nurses undertake important interventions that enhance the effectiveness of other healthcare services.

What is clear from current research is that intellectual disability nurses do not only work directly with people with intellectual disability, but more importantly play significant roles in the delivery of effective interventions by supporting other health and social care professionals who work directly with people with intellectual disability. To demonstrate that their interventions effectively meet the health, healthcare, and social care needs of people with intellectual disability, intellectual disability nurses need to provide evidence of the impacts of these interventions.

## Methods

### The survey method

By using the survey method, we were able to collect information on participants’ biographical information, countries in which participants practiced in and the types of organisations they worked for, patient (service user) group and interventions undertaken by the participants, case studies and examples of the impacts of the interventions undertaken by the participants, and participants’ understanding of intellectual disability nursing roles and interventions.

### Survey questionnaire development

We developed and piloted a survey questionnaire to identify intellectual disability nursing interventions, the impact of the interventions, and participants’ understanding of their roles. The questionnaire took approximately 30 minutes to complete and included 20 items across six domains: 1) biographical information (5 closed questions relating to age, sex, experience, qualifications, and registration status of participants; 2) countries and type of organisations (2 closed questions relating to the country, and type of organisation of participants; 3) patient (service user) group and interventions undertaken (6 questions (1 closed and 5 open ended) relating to age groups participants worked with, and nursing interventions undertaken by the participants); 4) examples of impacts of interventions (2 open ended questions relating to the needs of people given in the case studies); and 5) participants’ understanding of intellectual disability nursing roles (5 closed questions) (see Appendix 1).

### Sampling and participant recruitment

We set out to recruit intellectual disability nurses registered by the Nursing and Midwifery Council of the UK. However, we received responses from other intellectual disability nurses registered, as well as other registered nurses who exclusively worked with people with intellectual disability registered in other countries. Participants were therefore registered nurses and worked exclusively with people with intellectual disability. Ethics approval was obtained through the organisation of the lead researcher. Participants were recruited through professional networks. Participants were intellectual disability nurses or other nurses registered with a nursing regulator who worked exclusively with people with intellectual disabilities. Participants were recruited through intellectual disabilities professional networks. We used a combination of voluntary response sampling (McCombes, 2020) and snowball sampling (Creswell and Planko, 2017). Blending voluntary response sampling with snowball sampling or chain-referral sampling provided an opportunity for recipients of the survey link and or participants to share the survey link with their colleagues and intellectual disability nurses in their own professional networks (Creswell and Planko, 2017).

**Figure 1.**
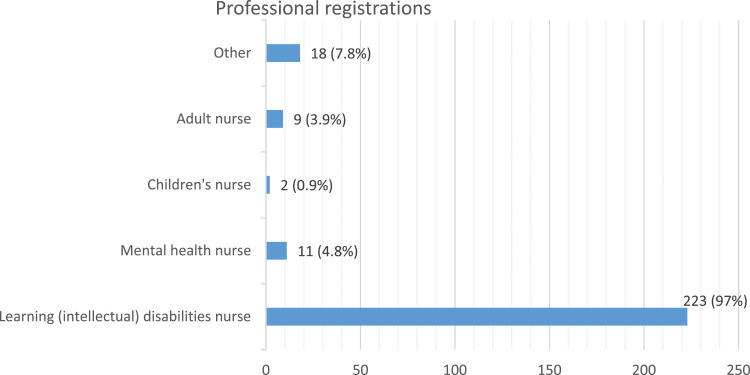
Professional registrations of participants.

**Figure 2.**
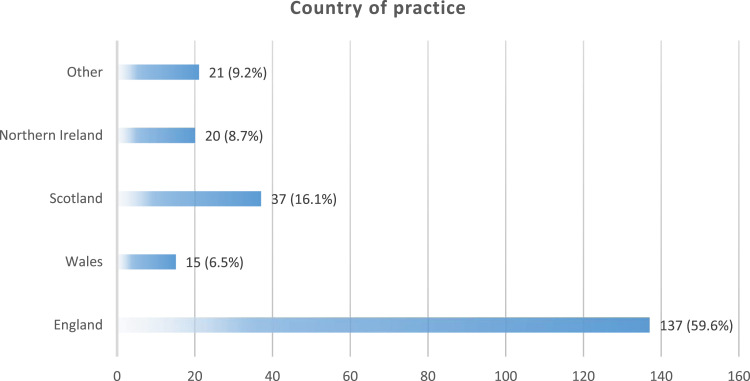
Countries where participants practice.

We used the G*Power sample size calculator (Heinrich State University, 2020) to ascertain the minimum sample required for the quantitative component of the study. The minimum sample size estimated was (*n* = 225). We collected data from *n* = 230 participants from seven countries (see Figures 1 and 2).

### Data collection

After ascertaining the content and construct validity of the survey questionnaire we created the online version on the JISC Online Surveys platform (https://www.onlinesurveys.ac.uk/). The survey was open from 13 October 2020 to 31 December 2020.

### Data analysis

Data analysis was undertaken using two distinct methods. In the first analysis we used thematic analysis to identify themes of the impact of intellectual disability nursing interventions using the Braun and Clarke (2006) methodology. In the second data analysis stage we used case study analysis method (Houghton et al., 2014) and identified numerous case examples of the interventions undertaken by intellectual disability nurses and organised these into 23 groups (see figure 2). Each theme was underpinned by several categories. For example; the Increased independence and choice theme was underpinned by 5 categories (*active involvement; informed decisions; making decisions; access to the community and other networks; independent*)

## Findings

We identified 13 interrelated themes of the impacts of intellectual disability nursing interventions (see figure 1). These are:

• Improved access to health and social care services
• Improved awareness of the needs of people with intellectual disabilities.
• Improved community presence and inclusion
• Improved family life
• Improved health and quality of life
• Improved healthcare outcomes
• Improved mental health and reduced challenging behaviour
• Improved standards, quality of care and patient experience
• Improved transitions
• Increased independence and choice
• Making reasonable adjustments
• Providing a voice
• Reduced health inequalities and risks

**Figure 3.**
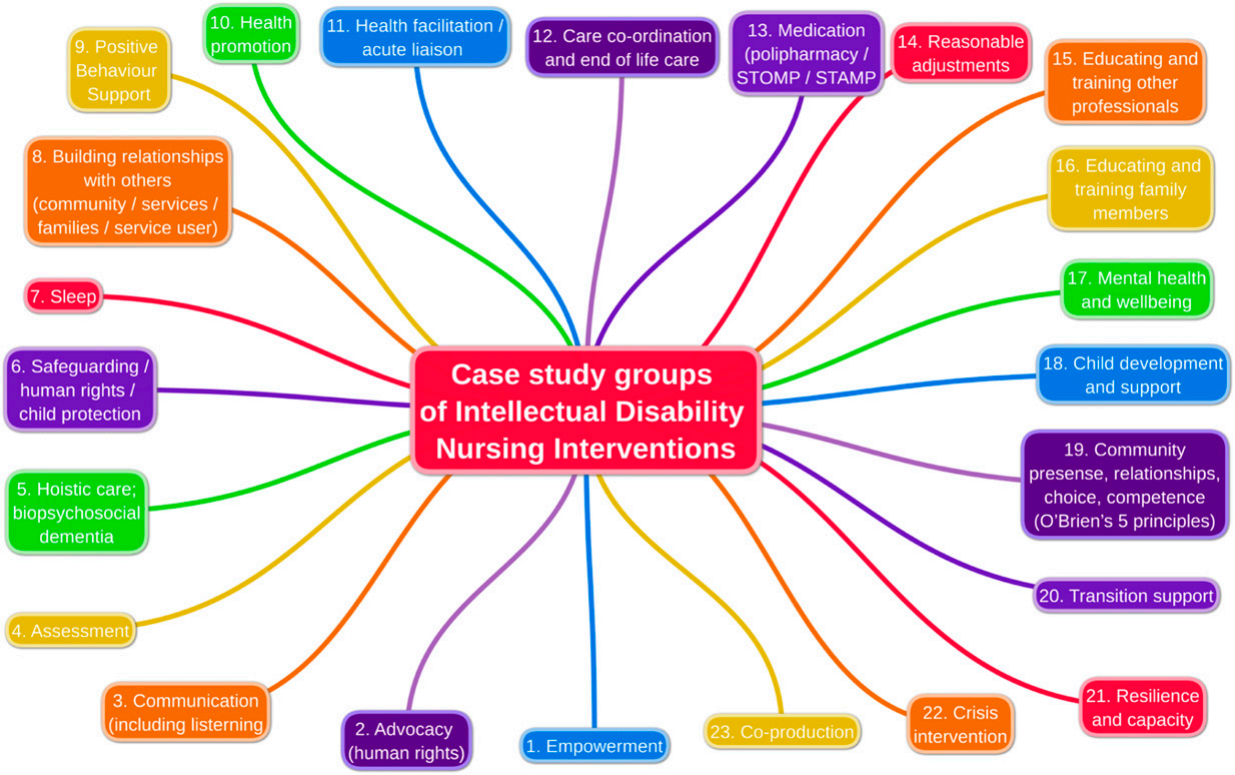
Impacts of intellectual disability nursing interventions.

In addition to the impacts of intellectual disability nursing interventions, we identified more than 100 case examples that we put into 23 broad groups of interventions that intellectual disability nurses identified that they carried out (see figure 3). These are:

• Advocacy
• Assessment
• Building relationships
• Care co-ordination
• Child development and support
• Co-production
• Communication
• Community presence
• Crisis intervention
• Educating and training family members
• Educating and training other professionals
• Empowerment
• Health facilitation
• Health promotion
• Holistic care
• Medication
• Mental health and wellbeing
• Positive behaviour support
• Reasonable adjustments
• Resilience and capacity
• Safeguarding
• Sleep
• Transition support

## Discussion

### Impacts of intellectual disability nursing interventions

**Figure 4.**
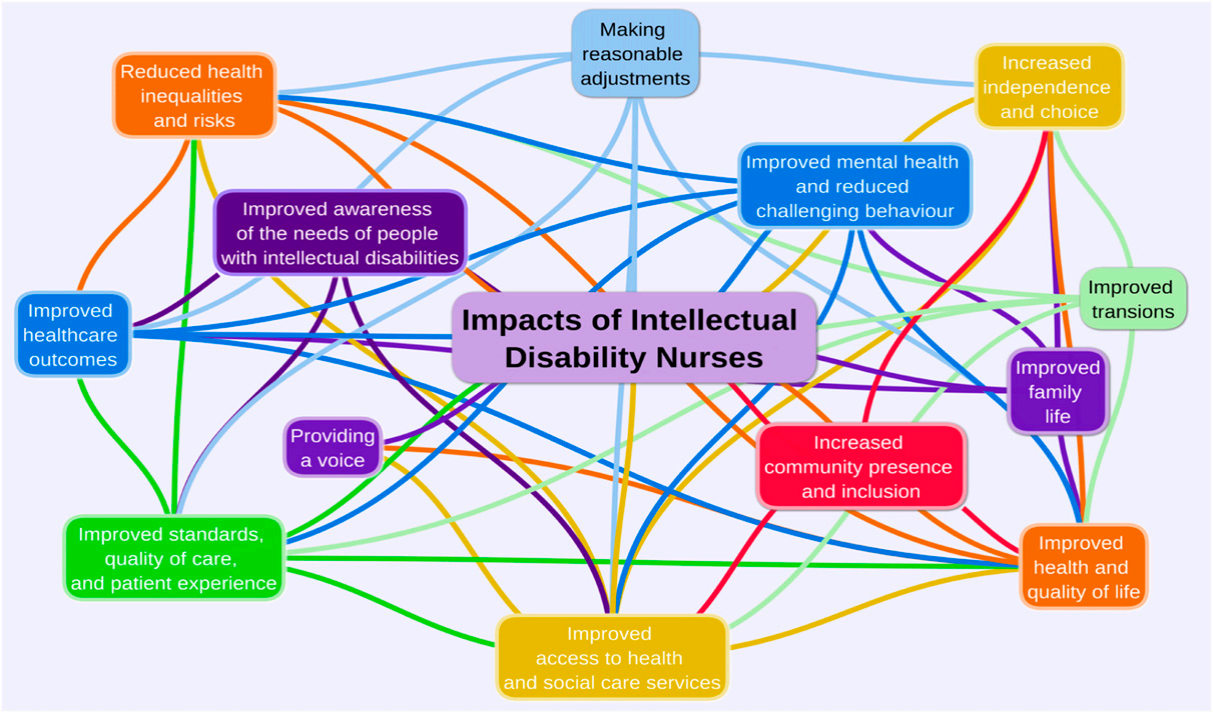
Case study groups of intellectual disability nursing interventions.

We asked participants in the survey to tell us the impact of their interventions on services and quality of life of people with intellectual disabilities. The impacts we identified related to intellectual disability nurses’ interventions with pregnant women, children, adults, older adults, and end of life. We u identified 13 themes (see Figure 4) of these impacts.

Intellectual disability nurses play an important role in problem solving and facilitating access to a wide range of health and social care services on two levels: directly for the services users, and indirectly by advocating for their families or carers. This advocacy ensures that professionals and services make appropriate decisions that result in positive outcomes for people with intellectual disability. These decisions resulting from intellectual disability nursing advocacy mean people with intellectual disability; receive appropriately funded care, adequate levels of support, equal access to services just like the general population, have their rights respected, and their families and carers receive appropriate support (Llewellyn and Northway, 2007).

As previously reported by Drozd and Clinch (216), intellectual disability nurse interventions improve the independence, and the ability to make choices by people with intellectual disability. The impact of this is that people with intellectual disability can make informed decisions about their health, healthcare, and lifestyles that result in improved and better quality of life. In addition, the ability to make appropriate and informed decisions is more likely to lead to improved quality of life in the community.

Preventative intellectual disability nursing interventions are central to improving the health and healthcare outcomes for people with intellectual disability. From the evidence from the participants in this survey, these interventions reduce health risks, improve the experience of care, improve physical and mental health, and reduce premature mortality among people with intellectual disability. The role of intellectual disability nurses in supporting people with intellectual disability to access appropriate services is longstanding (Robertson et al., 2015). What is apparent in the examples of impact provided here is of immense importance and significance to people with intellectual disability. The result of this is likely to improved access to services is better assessments treatments, and person-centred supports resulting in improved healthcare experiences and outcomes.

The standards and quality of care are central to service user experience when people need healthcare interventions. Poor quality healthcare leads to poor healthcare experience and outcomes. For people with intellectual disabilities this may result in premature mortality. Nursing interventions undertaken by intellectual disability nurses improve patient safety, make mainstream services better informed about the healthcare needs of people with intellectual disability, and ensures that people with intellectual disability have better experience of care (Taua et al., 2017; Lovell et al., 2016). The result is that people with intellectual disability may live longer lives in good health.

The health and healthcare needs of people with intellectual disability are complex and many mainstream services lack the awareness of these needs, and in many cases are unable to deliver the care that could be expected. Intellectual disability nursing interventions can support mainstream services to become more responsive to the complex needs of people with intellectual disability (Arrey 2014; McCarron et al., 2018). Intellectual disability nurses therefore have a key role in ensuring that healthcare staff, managers, and commissioners of healthcare services understand these complex needs.

People with intellectual disability experience health inequalities and inequities that are not experienced by people without intellectual disability (Chapman, 2015; Cope and Shaw, 2019; Kavanagh et al., 2017). This more often result in preventable illnesses, poor experiences of health and social care services, and some cases preventable early mortalities (Robertson et al., 2015; LeDeR, 2020). The interventions undertaken by intellectual disability nurses to reduce health inequalities and risk of illness and death are central to the health and healthcare outcomes for people with intellectual disability.

Many people with intellectual disability are unable to access the services they more often need than the general population (Brown et al., 2010). The reasons are multiple and include the fragmentation of health and social care services in the UK that may result in people with intellectual disability ‘*falling through the cracks*.’ The interventions undertaken by intellectual disability nurses are important in preventing people with intellectual disability from falling through these cracks.

People with intellectual disability often have long term or lifelong complex care needs (Brown et al., 2016; Pennington et al., 2019. Family support with all aspects of life is consequently a significant and important aspect of their lives. Examples of the case studies demonstrate that the interventions undertaken by intellectual disability nurses result in better supports for the families of people with intellectual disability. The interventions undertaken by intellectual disability nurses may result in improved family lives that may lead to improved and better outcomes for people with intellectual disability.

Because of complex health needs associated with intellectual disability, people with intellectual disability are known to need healthcare interventions more often. Healthcare professionals in mainstream services often do not have the necessary experience to understand the complexity of the healthcare needs of this population, resulting in diagnostic overshadowing that often results in unintended consequences (Taua et al., 2017). This study has shown that intellectual disability nurses play an important role that may result in more appropriate diagnoses and interventions that may result in reduced inpatient admissions, improved healthcare outcomes, and reduced suffering and even premature death. Therefore, the role of the intellectual disability nurse is central to the health and healthcare outcomes for people with intellectual disability.

### Case studies

Here we provide an overview of the groups of interventions and discuss 18 examples of the case studies. We acknowledge that it is not possible to provide details of all the case studies we identified in the study in a single paper. Here, we therefore provide only a selection of case examples from some of the 23 groups.

#### Empowerment (51 participants)

Under this theme, there was a consistent thread of promotion of autonomy, informed choice and using person-centred approaches to identify and support a positive life trajectory for people with intellectual disability. Service user groups included children and adults across all age groups including those with “challenging behaviour”, people with a diagnosed mental illness, people requiring palliative care, people with complex needs and people with undiagnosed or unmet health needs. Activities that incorporated this theme included: developing life plans; aiding with activities of living, including planning holidays and other activities with social capital (see *Case study 1*).

### Case study 1. Supporting adults who use wheelchairs with their activities of living

*“…promoting social-role valorisation, including planning a cruise holiday, clothes shopping for it, socialising and taking photographs and making scrapbooks as a topic of conversation…. The cruise had a] great impact on his mental health and wellbeing”*

Research has shown that empowered people with intellectual disabilities become more confident in in seeking help and support (Brown et al., 2016). Intellectual disability nurses play an important role in ensuring that people with intellectual disability are empowered to contribute to making important decisions about their lives.

#### Advocacy (32 participants)

There were many situations where intellectual disability nurses needed to act as advocates for the service users in their care. Women with intellectual disability may be seen as a risk to their own child when they are pregnant and come under the purview of child protection. When one intellectual disability nurse identified that the wording of a social services letter being sent to a pregnant woman about potential court proceedings and her baby being taken into care was negative, they advocated on behalf of the pregnant woman, explaining the impact of receiving such a letter in the post, resulting in changes to the wording and format (easy read). They delivered the letter with the social worker to explain the letter and the importance of the baby’s wellbeing, but also to highlight what was going well. The intellectual disability nurse carried out a similar role for another pregnant woman with a positive outcome (see Case study 2).

### Case study 2. Advocating for pregnant women

*“Pregnant lady with unborn child under child protection procedure. Education on pregnancy and parenting given. Acted as an advocate at child protection meetings, 1 year on and both parent and child are thriving together.”*

This case study demonstrates that advocacy, may be subsumed within many more concrete interventions, could drive the function of the intellectual disability nurse to save lives, to allow women to keep their children and look after them, to prevent the institutionalisation and deprivation of liberties that is a persistent threat to people with intellectual disability.

#### Communication (23 participants)

It is arguable that communication is fundamental to the delivery of healthcare care that it is almost assumed to be part of the role (Taua et al., 2017). In this study communication was identified as an intervention in their responses, from collaborative, active listening to developing services in this aspect of care.

The importance of modifying communication to meet the cognitive and self-actualisation needs of service users was also an important aspect of the work of the intellectual disability nurse (see Case study 3). This finding is consistent with recent studies, for example, Taua et al. (2017).

### Case study 3. Adapting communication

*“I listen to my clients and talk to them in a way that is suitable to the individual. I will do easy read literature and look for activities that they want to do, not what society says they can do”.*

Much of the time, communication is an assumed part of other interventions and nursing activities by the intellectual disability nurse, but these examples demonstrate the complexity of the skills used for communication and the centrality of adapted and individualised communication to meet a range of needs for the person with intellectual disability. Being supported by someone with expert listening and communication skills has a profound impact on the lives of people with intellectual disability. This is essential in ensuring development of easy-to-understand letters and provision of communication advice to other healthcare professionals (Lloyd and Coulson, 2014). Effective communication is essential for the provision of effective health care delivery.

#### Assessment (35 participants)

Some participants identified assessment amongst their interventions, including health assessment. The importance of the intellectual disability nurses’ role in health needs assessment has been previously highlighted (Pennington et al., 2019; Taua et al., 2017; Doody et al., 2019; Quinn and Smolinski, 2018). *One participant illustrated how* intellectual disability *nurses educate other health professionals to assess the health needs of people with* intellectual disability. Also included in assessment were identifying specific neurodiverse differences, such as autism.

Some health assessments were specific, for example, carrying out a continence assessment, or a pain assessment, while others were more generic and identified family assessment and working with families to assess the need for referrals to other agencies. Assessments were also discussed in terms of a holistic approach, to enable referrals to several different supportive specialists, for example speech and language therapists, and to co-ordinate the multi-professional team (see Case study 4).

### Case study 4. Assessing health needs

*“We work through observations, assessments, information gathering, pulling together multi-disciplinary teams, analysing and being creative in our strategies and recommendations to meet the individual needs of the patient and their families”.*

Assessment is a very broad term that captures a wide variety of specialist skills and knowledge across several different professional arenas and services. Intellectual disability nurses often use the terms holistic or biopsychosocial to indicate the importance of taking many factors into account when assessing any aspect of health and wellbeing for a person with intellectual disability, incorporating family, environmental cues and risks, functional analysis of behaviour, potential physical and mental health concerns, as well as potential safeguarding risks. This is essential to co-ordinate the input of other members of the multi-professional team and ensure it meets the specific and complex needs of people with intellectual disability. It is also essential in avoiding diagnostic overshadowing.

#### Holistic care (17 participants)

The importance of the role of the intellectual disability nurse in facilitating holistic nursing care has been previously highted (Doody et al., 2013). In this study some participants identified an aspect of their role as providing holistic or biopsychosocial care, a theme that was consistently discussed in relation to caring for people with intellectual disability and dementia and wherever support with achieving activities of living was a prominent aspect of care provision. It was also identified as an aspect of managing health needs (see Case study 5).

### Case study 5. Facilitating delivery of holistic care

*“We look at a person holistically and, using our contacts with other agencies, make necessary referrals to ensure reasonable adjustments are made to access appropriate health…”*

Dementia care was not only about providing direct care, but about supporting services to ensure care provision could be maintained when health became a concern, preventing placements from breaking down. Holistic care was also identified as important in identifying and meeting the needs of people with intellectual disability and mental ill health and people with epilepsy in the assessment and management of needs. Using a holistic approach to health care delivery to people with intellectual disability is an essential component of health facilitation to improve access to health care to improve outcomes and health care experience.

#### Safeguarding (23 participants)

Perhaps surprisingly to those who are not aware of the different skills in assessment acquired by the intellectual disability nurse, the need for safeguarding was evident even during acute hospital stays (see Case study 6).

### Case study 6. Safeguarding adults

*“She had been in the acute hospital for 9 days before I was informed of her admission, and they were getting ready to discharge her. I noticed she also has 2 fractured hips. The hospital had failed to identify that this should be an ASP [Adult Support and Protection] query. My input resulted in a big investigation happening with nursing home and patient being placed somewhere else. It was highlighted possible neglect where she had been residing also. This resulted in the patient being kept safe from potential abuse/harm and being treated with more dignity as she nears the end of her life.”*

This clearly highlights the importance of understanding the needs of people with intellectual disability from the perspective of an intellectual disability nurse with specialist knowledge and awareness of safeguarding issues. Nurses also identified safeguarding in relation to keeping service users with preventing people needing to be admitted to mental health facilities as well as maintaining their safety if they were admitted to acute services. Safeguarding is an important aspect of the role for those working with children and young people as well as in protecting people who were at risk of offending (McCarron et al., 2018). Awareness of the need for the person with intellectual disability to be protected from potential abuse is important in relationships involving people with intellectual disabilities. Adult safeguarding for people with intellectual disability require advanced knowledge and understanding of their potential needs and a broad range of expert skills in addressing them.

#### Positive Behavioural Support (PBS) (12 participants)

Forty participants in this study identified PBS interventions within their role. They reported working closely with families and paid carers to assess and plan interventions to reduce distress in people who exhibited “challenging behaviour”. One participant identified and discussed an important outcome of PBS in relation an adult who was assessed and treated for “challenging behaviour” that had led to a placement breakdown, was to shorten hospital stays and improve community settings and interventions (see Case study 7).

### Case study 7. Positive behaviour support (adult)

*“This made the period he spent in hospital shorter and ensured the new Care Team felt supported by staff with good knowledge and relationships with the young man while he settled into new placement. We could share PBS plans and advise what worked well with him and what had not. His transition back to the community was very successful and he continues to thrive.”*

The provision of positive behaviour support and training has been identified as an important intervention undertaken by intellectual disability nurses (Oulton et al., 2019). These interventions are significant to the health and healthcare outcomes of people with intellectual disabilities. It appears from the wide range of interventions reported in this study that increasingly, intellectual disability nurses are taking on new roles. This development is likely to improve how services respond to the healthcare needs of people with intellectual disabilities.

#### Health promotion (56 participants)

In this study health promotion was identified in numerous interventions, and, while often part of health liaison or health facilitation, it was also distinct from other activities such as health assessment, referrals, advocacy, support with engaging with tests and care interventions and care planning. Health promotion also involved teaching health care skills and self-monitoring to individuals with health needs, to enable them to manage their own health care needs. These interventions are consistent with previous studies, for example, McCarron et al. (2018), Northway et al. (2017) and Doody et al. (2019).

In this study intellectual disability nurses demonstrated how they delivered health promotion in different ways (see Case study 8).

### Case study 8. Promoting health

*“An individual had a high BMI, type 2 diabetes - did not have a healthy balanced diet when attending day-care. Lunch would have included 3 packets of crisps and 4 bars of chocolate. I delivered health promotion sessions to the group room - which included healthy eating, active lifestyles, diabetes. Through regular health promotion, this service user gradually reduced the amount of crisps, bars etc. His weight and BMI had reduced.”*

#### Health facilitation and liaison (48 participants)

Previous studies have demonstrated the importance of health liaison and health facilitation activities (Northway et al., 2017; Chapman, 2015). In this study, health facilitation constituted the most frequently identified set of interventions for intellectual disability nurses, with many being involved in health assessments and referrals, as well as supporting with attendance at and tolerance of appointments for examinations, reviews, tests, and treatments, including one where a woman had been referred because of behaviour that was a cause for concern in day services, preventing her from attending. One participant described a family that was struggling to cope. They described how they supported the person with intellectual disability with all their health appointments including their annual health check and specialist dentistry referral and optician review. Their health needs were assessed, investigated, and met – with the intellectual disability nurse supporting them until they were able to tolerate a full dental check (see Case study 9).

### Case study 9. Health liaison

*“…. she had 17 rotten teeth, abscess, and infection. All rotten teeth extracted, and antibiotics given for infection. Supported with aftercare, and behaviours stopped at day service.”*

The health facilitation and health liaison interventions undertaken by intellectual disability nurses appear to be expanding at pace. For people with intellectual disabilities, these interventions may mean the difference between accessing appropriate mainstream services and support. To improve services and enhance their impact intellectual disability nurses need to work collaboratively to improve access to mainstream services as well as take up direct care roles in mainstream services. The introduction of the Future nurse (NMC, 2018) standards in the United Kingdom places future intellectual disability nurse graduates in a unique position to assimilate these emerging roles in mainstream services.

#### Care co-ordination (75 participants)

The role of the intellectual disability nurse in co-ordinating care across a wide range of settings has been demonstrated to be essential in facilitating delivery of effective care to people with intellectual disabilities (Cope and Shaw, 2019; MacArthur et al., 2015). In this study intellectual disability nurses described how by focussing on the individual and their needs, they worked across a range of services at operational as well as system level (see Case study 10).

### Case study 10. Making referrals

*“Assessed her continence needs in the first instance and checked she didn't need products prescribed and how much of the issue she understood. Identified training needs for the service and delivered bespoke training for them in continence, constipation, and dignity in care. Advised the commissioners and CQC that they require their contracts to be looked at due to confusion around what 'care' they can and cannot deliver. Screened the young lady for any underlying health issues and ensured all health needs are being met - some minor things identified from our initial screening that have since been answered e.g., dental care, orthotics and epilepsy review - intervened with these where appropriate. Referred to community intellectual disability occupational therapy for further assessment around toilet routine and they are now working directly with her to increase her independence and skill set in this area, with the hope she can return to day services.”*

It is evident from this evidence that intellectual disability nurses undertake important interventions in enhancing the impact of mainstream services and healthcare professionals who work in these services. For example, working with mainstream services to put reasonable adjustments in place, and training mainstream staff regarding the needs of people with intellectual disabilities. This is important because intellectual disability nurses need to support other healthcare professionals who work directly with people with intellectual disabilities across the lifespan.

#### Medication (66 participants)

The nurse’s role in medication prescribed to people with intellectual disability was discussed in multiple ways in this study and has been previously highted by, for example, Adams and Shah (2016). Participants in this study described supporting individuals to access, manage, and understand their needs as well as ensuring that appropriate prescribing and review patterns were supported with clinicians was highlighted in the case studies. The below example highlights assessment, monitoring, review, and collaborative working with professionals as well as families and carers to ensure a therapeutic plan for the person and generalisation of knowledge across environments. This level of intervention relates directly to NHS England’s principles of Stopping over medication of people with a learning disability, autism or both (STOMP) with nurses clearly engaged in these agendas (see Case study 11).

### Case study 11. Medication (assessment and monitoring)

*“An individual with food intolerance and changing bowel habits for a year. Implemented bowel charts and spoke with parents for assessment. Took information to GP with concerns of constipation. GP prescribed laxative. Through monitoring we achieved a therapeutic dose for the individual which allows them to have regular bowel movements. Throughout this I supported parents and provided information on the medication to reassure their concerns. The individual now has a bowel management plan in place involving parents, college, and respite.”*

Diagnostic overshadowing was identified, and associated with medication prescribed, and the ongoing effects of these for individuals. Educating people and their families and carers appears fundamental to the intellectual disability nurse’s role in ensuring that medication is appropriate and understood.

#### Reasonable adjustments (56 participants)

Flagging and making reasonable adjustments have been previously identified and described as essential interventions performed by intellectual disability nurses to improve access to, and the quality of health care for people with intellectual disabilities (Oulton et al., 2019; Doody et al., 2019; Sheerin, 2012). In this study facilitating reasonable adjustments was one of the most prevalent interventions undertaking taken by intellectual disability nurses across the lifespan and in a wide range of settings. Participants described the importance of ensuring that the people they supported had equality of access through the application of the Equality Act 2010 in the UK. The interventions were described in multiple examples of ensuring people’s health care needs were met, that individuals could access appropriate information and give consent in an informed and supported way (see Case study 12).

### Case study 12. Supporting others and making reasonable adjustments

*“I make a difference by helping all those who support the person to understand the unique nature of their needs and all the reasonable adjustments big and small, in a physical sense and a theoretical sense, that we have to make to even begin to make my client’s access to health and wellbeing on a par to that of the wider population.”*

The making or facilitation of the making of reasonable adjustments by intellectual disability nurses is essential in ensuring that other health care professionals can deliver person centred care that benefit of individuals with intellectual disabilities.

#### Educating and training (16 participants)

The role of the intellectual disability nurse in training staff teams and services was a common intervention discussed in this study as in previous studies (Cleary and Doody, 2017). The importance of training, and the involvement of people with intellectual disability and / or autism is important as described in in the below case study (see Case study 13). Supporting individuals to undertake this important role and working in true partnership towards co-production demonstrates the power of the intellectual disability nurse in role modelling best practice (see Case study 13).

### Case study 13. Staff training

*“….is to provide training to services which support people with an LD to help them understand how autism can affect people and look at ways of supporting people. This has always been done with myself and an autistic person providing the training so they can articulate their experiences and answer any questions which is very helpful …”*

Facilitating acquisition of knowledge, understanding of function and the acquisition of new is an important area of intellectual disability nursing. Educating staff to better support people with intellectual disabilities to make their own choices (Sheerin, 2012), and educating people with intellectual disabilities and their families and carers about health and healthy lifestyles and how to cope with diagnoses and symptoms (Cleary and Doody, 2017) are important interventions undertaken by intellectual disability nurses. The extent of these interventions clearly demonstrate that intellectual disability nurse interventions are wide ranging across the lifespan.

#### Mental health and wellbeing (21 participants)

The intellectual disability nurse’s role in supporting people’s mental health needs was discussed across multiple interventions and has been previously described by Taua et al., 2017). In this study general mental wellbeing was a recurrent theme described by participants to identify trauma, anxiety, and distress experienced by people with intellectual disabilities. Intellectual disability nurses play an important role ensuring that people can maintain their relationships with mainstream services (see Case study 14).

### Case study 14. Assessing mental health

*“I have recently supported someone experiencing a decline in their mental health. Through appropriate support and advice, we were able to prevent hospital admission. Through the use of person-centred care and reasonable adjustments. The result of this has been person still has trust in myself and services and is still engaging.”*

Support during a mental health crisis was described within interventions associated with transitional care arrangements between environments, providing accessible information and the need to ensure care is person centred. Education and liaison with services is key to this role ensuring long term sustainable solutions for people with an intellectual disability and mental health issues (see Case study 15).

### Case study 15. Supporting mental health recovery

*“I met the patient and supported admission to the local mental health unit on the same day. I visited regularly whilst she was an inpatient to build a relationship and I continue to work with her now that she is home. I have been providing education around trauma and how these experiences still affect her, coping/redirection and grounding strategies, mental health monitoring, supporting contact with GP around physical health issues as required and emotional support.”*

#### Child development and support (7 participants)

Working alongside children and young people and their families and carers is a key role of the intellectual disability nurse (Cope and Shaw, 2019; McCarron et al., 2018; Ring et al., 2018). Taking a holistic view of a child can facilitate collaborative working across services to enable and support treatment, development, and appropriate developmental care. Describing care that had been based on an inappropriate diagnosis the below intervention enabled a young person to move forward with appropriate care based on child centred outcomes (see Case study 16).

### Case study 16. Collaborating holistic care (children)

*“(GP/A&E/social worker/school staff) believing this to be psychosis …. Over time use of medication and both narrative and EMDR therapy to help process the trauma and help (with carers) to reduce fear and regain skills and build new relationships.”*

Working with children and young people for the intellectual disability nurse presents an opportunity to co-ordinate family centred care that can support real change in their ability to meet the needs of their child over time through developmental and transitional changes.

#### Transition support (10 participants)

Interventions focused on supporting transition for individuals with an intellectual disability featured across many of the case studies described by nurses. Transition across services, changes in provision, age related service changes were all described as part of the care that nurses co-ordinated for individuals. For one nurse working within an acute care setting transition was described as part of her role in ensuring health needs were met across departments and between appropriate hospitals (see Case study 17).

### Case study 17. Supporting transitions to adult services

*“I support transition to adult services, inpatients, outpatients and elective surgery patients with intellectual disabilities and / or autism to ensure their contact with the trust is of a high standard, which meets their additional needs and ensures they get an equitable service.”*

This is important and we concur with that Interventions by intellectual disability nurses are essential in enhancing the effectiveness of transition services (Delahunty, 2017). Intellectual disability nurses are often in more regular contact with children, they support and therefore better placed to facilitate links between services. Increasingly, in our own experiences more intellectual disability nurses are taking on roles in school nursing services. This development is likely to improve how services respond to the healthcare needs pf children with intellectual disabilities.

#### Resilience and capacity building (4 participants)

This theme considered the nurse’s role in supporting and building the resilience and capacity of those supporting individuals as well as people with an intellectual disability. Participants described this intervention in relation to services, families and staff and was considered as fundamental to the role of the nurse in facilitating holistic care. Considering the complex needs of a person who required extensive medical interventions one participant described the wider role required to enable person centred care to be provided (see Case study 18).

### Case study 18. Capacity building

*“My involvement in facilitating other professionals to be involved, liaison with GP supported this service user having as many investigations as possible whilst under GA [general anaesthetic]. I ensured everyone talked to each other and reiterated useful information about presentation of the person to help decision making. I supported the family and carers emotionally and reassured staff who were unsure on next steps when plan did not work in a straightforward way. Flexibility in approach and knowledge supported the process and this service user had only one GA for multiple examinations and investigations to help maintain his health.”*

Intellectual disability nurses enable the provision of holistic care associated with increased capability due to deeper understanding of the individuals they support (Llewellyn, 2005). For families and carers, the role of the nurse in the provision of interventions was evident throughout the case studies. The importance of the roles of intellectual disability nurses in supporting other services to support people with intellectual disabilities better cannot be overemphasised. Intellectual disability nurses practice in complex environments which, are often multi-disciplinary and multiple agencies. This will require them to engage in creative communication to enable things to happen. Furthermore, there is a need for intellectual disability nurses to provide leadership in improving health care services through troubleshooting and other interventions.

## Conclusions and implications for practice

Intellectual disability nurses carry out many interventions that positively impact outcomes for people with intellectual disability, including safeguarding, positive behavioural support, promoting mental wellbeing, advocacy and empowerment, leadership, liaison with other agencies, educating and supporting other staff and families, assessment, and monitoring of appropriate medication. Awareness of the roles of intellectual disability nurses and their importance in addressing health inequalities and facilitating the use of mainstream services for people with intellectual disability will enable improved outcomes for people with intellectual disability.

The case studies in this research demonstrate that intellectual disability are often working at an advanced practitioner level and frequently need to be involved in care delivery by other practitioners to ensure that health and social care, including engagement with legal frameworks, is adapted, and augmented to meet the needs and human rights of people with intellectual disabilities. What is also clear from this research is the wide range of interventions that intellectual disability nurses undertake in a complex sphere of practice. It is clear from the extent of these interventions that intellectual disability nurses need to constantly adapt and engage in a wide range of roles, and that they need to constantly assimilate emergent roles (Northway et. al., 2017). What also emerges from this research are the complexities and changing needs of people with intellectual disability, the changing environments in which intellectual disability nurses are practising, and the increasing expectation for intellectual disability nurses to meet health, healthcare, and social needs of people with intellectual disability across the lifespan.

The World Health Organisation’s policy on disability (WHO, 2020) advocate for equitable access to effective health services and public health interventions by people with intellectual disabilities, to achieve the highest attainable standards of health. Changes to pre-registration nursing standards in the United Kingdom in 2018 now require intellectual disability nurses to be proficient with a new array of nursing skills that were previously only associated with the adult nursing field of practice. These new proficiencies mean that intellectual disability nurses will be proficient in performing more nursing procedures that were previously undertaken by other nurses. These are welcome developments that are likely to result in better care and better outcomes for people with intellectual disabilities.

We recommend that further work be undertaken to promote and publicise the impact and contributions of intellectual disability nurses. Importantly we recommend that some of this work focus on the unique contributions made by intellectual disability nurses to mainstream services and agencies. This is important for planning and resource allocation for future service provision.

## Limitations

Although our findings are not generalisable and need to be understood from the context of participants who took part in the study, they provide an important starting point for future work. Also, it is important to note that the participants self-selected and therefore data may be biased. Covid-19 restrictions impacted on the process of data collection, and this affected our ability to verify some of the case studies and examples of the impacts that were reported.

## Supplemental Material

Supplemental Material - Understanding the contribution of intellectual disabilities nurses. Paper 4 of 4 - Impacts of intellectual disability nursing interventions Supplemental Material for Understanding the contribution of intellectual disabilities nurses. Paper 4 of 4 - Impacts of intellectual disability nursing interventions by Kay Mafuba, Dorothy Kupara, Hazel M Chapman, Rebecca Chester, Joann Kiernan, and Chiedza Kudita in Journal of Intellectual Disabilities

## Data Availability

Raw data were generated at the University of West London. Data supporting the findings of this study are available from the corresponding author Professor Kay Mafuba (kay.mafuba@uwl.ac.uk) on request. Data will be retained for a minimum of 5 years.
