# Supplementary material for: Understanding the contribution of intellectual disabilities nurses. Paper 4 of 4 - Impacts of intellectual disability nursing interventions
Source: J Intellect Disabil. 2024 Jan 18;30(2):243–65. doi: 10.1177/17446295241228044 (PMC13230655; doi:10.1177/17446295241228044)
Supplement: Supplemental Material - Understanding the contribution of intellectual disabilities nurses. Paper 4 of 4 - Impacts of intellectual disability nursing interventions [file sj-pdf-1-jld-10.1177_17446295241228044.pdf]

Appendix 1 – Survey questionnaire

About me

1 How old are you?

1. 20-30 years
2. 31-40 years
3. 41-50 years
4. 51-60 years
5. Over 60 years

2 Gender

1. Female
2. Male
3. Do not wish to say

3Years of experience as a registered intellectual (intellectual) disabilities nurse

1. Less than 5 years
2. 6-10 years
3. 11-20 years
4. 21-30 years
5. More than 30 years

4 My highest qualification

1. Diploma
2. Degree
3. Post Graduate Diploma
4. Master's Degree
5. PhD or other research degree
6. Other

a If you selected Other, please specify:

5 I am registered with the professional body as (Select all that applies);

1. Intellectual / intellectual disabilities nurse
2. Mental health nurse
3. Children's nurse
4. Adult nurse

Other (Please specify)

a If you selected Other, please specify:

Where I work and what I do

6 Country where I practice

1. England
2. Wales
3. Scotland
4. Northern Ireland
5. Other

a If you selected Other, please specify:

7 Type of organisation I work for

1. NHS (LD – Inpatient)
2. NHS (LD – Community)
3. NHS (Acute Hospital)
4. Local authority
5. Private / Voluntary organisation
6. School or other children's service
7. Higher education or research
8. Other

a If you selected Other, please specify:

8 The age group(s) of people I work with (Select all that applies)

1. Maternity (pregnant women)
2. Children (0-17 years)
3. Adults (18-65 years)
4. Adults (Over 65 years)
5. Palliative / End of life care (could be any age)
6. Other

a If you selected Other, please specify:

9 List all nursing procedures you undertake (these are activities you practically do, e.g., administering medication; managing aggression).

10 List all activities you undertake to improve how other services or professionals deliver effective care to people with intellectual disabilities (e.g., making reasonable adjustments).

11 List all activities you undertake to improve the quality of life of people with intellectual disabilities (e.g. health promotion).

12 List any other activities you undertake you have not described above.

13 Tell us the impact of what you do on services and quality of life of people with intellectual (intellectual) disabilities.

Example(s) of what I/we do which makes a difference

14 Describe the person (people) and their need(s). Tell us what you did (do) which made (makes) a difference? How did (does) what you do make a difference to the person (people) you support?

15 We would really want to get more information on what you do. If you would like us to contact you please provide us with details (Name and E-mail address)

What you think about what intellectual (intellectual) disability nurses do.

16 How confident do you feel in your understanding of what all intellectual (intellectual) disabilities nurses do?

1. Not at all confident
2. A little confident
3. Very confident

17 How confident do you feel in your understanding of all the nursing procedures intellectual (intellectual) disabilities nurses are expected to perform in roles?

1. Not at all confident
2. A little confident
3. Very confident

18 How confident do you feel in your understanding of all activities intellectual (intellectual) disabilities nurses are expected to undertake in order to improve how other services or professionals deliver effective care to people with intellectual (intellectual) disabilities?

1. Not at all confident
2. A little confident
3. Very confident

19 How confident do you feel in your understanding of all activities intellectual (intellectual) disabilities nurses are expected to undertake in improving the quality of life of people with intellectual (intellectual) disabilities?

1. Not at all confident
2. A little confident
3. Very confident

20 A compendium describing what intellectual (intellectual) disabilities nurses do would be a useful resource for my practice.

1. Strongly disagree
2. Disagree
3. Not sure
4. Agree
5. Strongly agree

Final page

That was the last question. We want to thank you again for taking time to participate in this research. We will share with you the findings of this research.
